# Relationship of oxytocin and cortisol response to psychosocial stress in children and adolescents with anxiety disorders

**DOI:** 10.1038/s41598-026-44831-8

**Published:** 2026-03-26

**Authors:** Leonie Goetz, Irina Jarvers, Daniel Schleicher, Angelika Ecker, Inga D. Neumann, Romuald Brunner, Stephanie Kandsperger

**Affiliations:** 1 Department of Child and Adolescent Psychiatry and Psychotherapy, University of Regensburg, Regensburg, Germany; 2 Department of Behavioural and Molecular Neurobiology, Regensburg Center of Neuroscience, University of Regensburg, Regensburg, Germany

**Keywords:** Cortisol, Oxytocin, Stress response, Adolescents, Recovery, Anxiety, Diseases, Neuroscience, Psychology, Psychology

## Abstract

Anxiety disorders are the most common psychiatric disorders in childhood and represent a high risk for adult mental disorders. The neurocircuitries of anxiety are known to interact with the neurocircuitry of stress. The neuropeptide oxytocin is known for its anxiolytic and antistress effects. This study is the first to examine oxytocin release in response to psychosocial stress in children and adolescents with anxiety disorders while monitoring the reactivity of the hypothalamic-pituitary-adrenal (HPA) axis. Altogether 32 adolescents with anxiety disorders and 32 healthy adolescents (aged 11–18 years) completed the Trier Social Stress Test. We measured salivary oxytocin and cortisol, subjective stress ratings, and heart rate. Salivary oxytocin and cortisol concentrations increased significantly after stress exposure, with no significant group differences. Pre-stress oxytocin levels positively correlated with cortisol levels in all participants during recovery. Healthy adolescents had higher levels of cortisol recovery. At all measurement points, participants with anxiety disorder reported significantly higher subjective stress and anxiety levels than the healthy controls. Across all participants, higher levels of cortisol and oxytocin recovery were associated with lower subjective stress levels during recovery. Oxytocin emerged as a consistent stress marker regardless of the presence of anxiety disorder, indicating its importance in post-stress recovery and subjective stress experience. Future studies are needed on the interactions between oxytocin and HPA axis in the context of psychopathologies.

## Introduction

Anxiety disorders are the most common psychiatric disorders in childhood^1^, with a lifetime prevalence of 15 to 20%^2^. Anxiety disorders in childhood and adolescence represent a high risk for adult mental disorders and often co-occur with other psychiatric disorders, like depression^3^. Given the high prevalence in children and adolescents and the substantial impairment of quality of life^4^, a better understanding of the pathophysiology and etiology of anxiety disorders is needed. Although candidate genes, environmental risk factors^5^, behavioral traits, and brain activity patterns^6^ may play a role in the development of anxiety disorders, neuroendocrine factors are of particular interest in the pathogenesis of these disorders. Anxiety neurocircuitries interact and overlap with the neurocircuitry of the stress response at various brain levels^7^. A better understanding of endocrine functioning in the neurocircuitries of anxiety and fear^7^ may enhance pharmacological treatment options^8–10^.

The neuropeptide oxytocin has gained attention in clinical neuroscience due to its role in stress regulation and stress-related disorders, including depression and anxiety disorders^8,10–12^. Beyond its reproductive and prosocial effects^13^, numerous animal and human studies have indicated antistress and anxiolytic effects of oxytocin^8,10,11,14^. Oxytocin is primarily synthesized in magnocellular neurons, specifically in the supraoptic and paraventricular nuclei of the hypothalamus, and released along the axonal projections from the posterior lobe of the pituitary gland into the peripheral circulation upon stimulation (e.g., social-emotional stress, physical exercise)^9,11^. In addition to release from axonal terminals, there is dendritic oxytocin release into the extracellular fluid, explaining basal and stimulated levels^9,15^. Oxytocin acts as a neuromodulator and neurotransmitter in Oxytocin Receptor-expressing brain regions^9^ as well as peripheral organs^11^. Oxytocin can be measured in blood plasma, cerebrospinal fluid, urine, as well as saliva, reflecting either the peripheral or central oxytocin system^16^. Central oxytocin and peripheral oxytocin can be released independently or simultaneously^11^. Since saliva samples are a simple, non-invasive method that does not require medical care, they are often used in clinical research to quantify oxytocin release before and after stimulation as a marker of global oxytocin system functioning^11,16^. Thus, studies with healthy adults have shown increased oxytocin concentrations in plasma and saliva during physical exercise (e.g.^16^), and psychosocial stress, such as in the Trier Social Stress Test (TSST)^16–20^. Bernhard et al. replicated these findings with increased salivary oxytocin release after the TSST in healthy children and adolescents^21^. Blunted oxytocin stress response has been demonstrated in youths with major depressive disorder^22^, conduct disorder^23^, or borderline personality disorder^24^. Overall, oxytocin reactivity to psychosocial stress has been investigated rarely in adults and adolescents with mental disorders, particularly none on anxiety disorders, so far. Recent evidence from animal studies and studies with human adults suggests dysfunction of the oxytocin system in psychiatric disorders associated with socio-emotional deficits, such as anxiety disorders^9,10,12^. Neumann and Slattery hypothesized that low oxytocin brain activity is associated with high anxiety levels, whereas a high central oxytocin availability seems to be associated with an anxiolytic effect^10^. Considering that mental disorders often emerge in childhood and early experiences influence the adult oxytocin system^12^, it is surprising that the oxytocin stress response in anxiety disorders has not been studied in children and adolescents, so far. Besides the physiological stress response, the TSST induces a psychological stress response, such as an increase in perceived stress and anxiety^25^. In a study by Tabak et al. with healthy adults, participants with high social anxiety before the test session showed a greater increase of state anxiety induced by the TSST compared to the adults with low social anxiety^20^. Social anxiety was associated with a larger post-stress increase in oxytocin levels^20^. In clinical samples like youth with major depressive disorder^22^ or borderline personality disorder^24^, they reported significantly higher TSST-induced subjective stress and anxiety levels compared to healthy controls, but blunted oxytocin release. In a non-clinical sample of children and adolescents, peripheral oxytocin levels increased in response to the TSST and baseline oxytocin levels negatively correlated with experienced anxiety in stressful situations^21^. In addition, participants with a primarily low basal oxytocin level had higher oxytocin release during the TSST^21^.

Given cortisol’s key role in stress regulation, reflecting the activity of the hypothalamic-pituitary-adrenal (HPA) axis, and the role of oxytocin in regulating the HPA axis^26^, this study investigated the interplay between cortisol and oxytocin in children and adolescents with anxiety disorders compared with healthy controls. We hypothesized that oxytocin plays an essential role in HPA dysfunction associated with psychopathology^27^. Previous studies^16,19,28–30^ in adults have suggested regulatory relationships between oxytocin and cortisol, with controversial findings. Some studies associated increased oxytocin levels with decreased cortisol release, leading to increased calmness and reduced anxiety^28,29^, whereas others noted positive correlations between oxytocin and cortisol during stress^16,19^. In addition, some studies have reported a blunted cortisol response to the TSST in adults with anxiety disorders, hinting at a HPA axis hypo-responsiveness^31,32^. Alley et al. proposed that these contradictory results stem from different time scales of the cortisol-oxytocin relationship^18^. In their study, women with higher oxytocin reactivity after stress exposure had greater decreases in cortisol during the recovery period, emphasizing oxytocin’s capacity to regulate post-stress recovery of the HPA axis^18,19^. In healthy children and adolescents, a moderate positive correlation between oxytocin and cortisol increase, as well as oxytocin increase and cortisol recovery, was found^21^. However, only one study examined oxytocin and cortisol release under psychosocial stress in a clinical sample of youth with major depressive disorder^22^, where no correlation between oxytocin and cortisol was found. In youth with conduct disorder, no correlation between oxytocin stress reactivity and cortisol reactivity was found either^23^. To the best of our knowledge, no study in a clinical sample of children as well as adults with anxiety disorders investigating the correlation between cortisol and oxytocin under psychosocial stress conditions exists so far.

In a controlled, experimental, observational study, we monitored the reactivity of the oxytocin system and HPA axis under basal conditions and during stress in children and adolescents (aged 11–18 years) with anxiety disorders. Because of the stress-buffering effect of oxytocin^10^, we expected the clinical sample to show a lower basal oxytocin level before stress exposure and less oxytocin reactivity than healthy controls after psychosocial stress induction. The levels of perceived feelings of anxiety and stress were also expected to differ significantly between both groups and to correlate negatively with oxytocin levels. We further hypothesized that lower oxytocin release after stress correlates with a higher increase in cortisol and a higher subjective stress response.

## Methods and materials

### Study design and recruitment

Our single-center, experimental, observational study with a matched control group involved 32 children and adolescents (aged 11 to 18 years) with anxiety disorders (based on DSM-5 and ICD-10 criteria) and 32 healthy same-aged controls (*N* = 64). Participants with anxiety disorder were recruited at the Clinic for Child and Adolescent Psychiatry, Psychosomatics, and Psychotherapy at the University of Regensburg, Germany. Healthy participants were recruited via study flyers, email distribution to colleagues, and advertisements on the clinic website.

The study was preregistered in the German Clinical Trials Register on 11 September 2019 (DRKS00017793), and the study protocol was published previously^33^.

### Study participants

As described in detail in the study protocol^33^, the main inclusion criterion for the anxiety group was meeting the diagnostic criteria for anxiety disorder according to the ICD-10 and DSM-5 (Table 1). Exclusion criteria were IQ < 85, pubertas praecox vera, pregnancy, known genetic syndromes, history of traumatic brain injury or endocrinological disorder, current glucocorticoid-containing medication, any other chronic neurological disorder, acute suicidal behavior, and any other current or lifetime history of psychiatric disorders except mild or moderate depressive episodes (ICD-10: F32.0, F32.1, F33.0, F33.1). For healthy controls, additional exclusion criteria were a history of or any current psychiatric disorder, as well as any psychiatric/psychotherapeutic treatments.

**Table 1:** Sociodemographic, clinical characteristics and physiological measures.

Variable		Anxiety group		Control group		Group comparisons^1^		
Age (in years)		M	SD	M	SD	U	*p*	*r*
		15.38	1.41	14.75	2.00	406.50	0.15	0.18
Sex*		***N***	**%**	***N***	**%**	***U***	***p***	***r***
Female		21	65.6	13	40.6	384	0.05	0.25
Male		11	34.4	19	59.4			
**School type**		***N***	**%**	***N***	**%**	***U***	***p***	***r***
Lower secondary		7	21.9	2	6.3	432	0.24	0.15
Intermediate secondary		5	15.6	5	15.6			
Academic secondary (college-preparatory)		15	46.9	20	62.5			
Other		2	6.3	2	6.3			
None		3	9.4	3	9.4			
Pubertal status**		***N***	**%**	***N***	**%**	***U***	***p***	***r***
Early Pubertal		-	-	8	25.0	326	0.01	0.33
Midpubertal		6	18.8	9	28.1			
Late Pubertal		14	43.8	7	21.9			
Postpubertal		12	37.5	8	25.0			
**Medication**		***N***	**%**	***N***	**%**			
Yes		12	37.5	2	6.3			
No		20	62.5	30	93.8			
**Hormonal contraception**		***N***	**%**	***N***	**%**			
Yes		3	9.4	1	3.1			
No		29	90.6	31	96.9			
**Level of intelligence**		***N***	**%**	***N***	**%**	***U***	***p***	***r***
Average (85–114)		20	62.5	17	53.1	486.50	0.70	0.05
Above average (115–129)		9	28.1	15	46.9			
Far above average (≥ 130)		3	9.4	-	-			
BAI***		***M***	***SD***	***M***	***SD***	***U***	***p***	***r***
		23.50	13.67	4.59	5.45	41.50	< 0.001	0.79
BDI-II***		***M***	***SD***	***M***	***SD***	***U***	***p***	***r***
		24.25	12.76	2.63	4.01	23.50	< 0.001	0.82
SPAIK***		***M***	***SD***	***M***	***SD***	***U***	***p***	***r***
		29.89	12.44	6.12	6.02	56	< 0.001	0.77
**Anxiety diagnoses (ICD-10)**		***N***	**%**	***N***	**%**			
F40.01	Agoraphobia with panic disorder	3	8.3	-	-			
F40.1	Social phobias	26	72.2	-	-			
F41.0	Panic disorder	2	5.6	-	-			
F41.1	Generalized anxiety disorder	3	8.3	-	-			
F93.0	Separation anxiety disorder of childhood	1	2.8	-	-			
F93.80	Generalized anxiety disorder of childhood	1	2.8	-	-			
**Comorbid depression diagnoses (ICD-10)**		***N***	**%**	***N***	**%**			
F32.0	Mild depressive episode	6	18.8	-	-			
F32.1	Moderate depressive episode	19	59.4	-	-			
F33.1	Recurrent depressive episode, current episode moderate	3	9.4	-	-			
None		4	12.5	-	-			
**Cortisol (in nmol/l)**		***M***	***SD***	***M***	***SD***	***t***	***p***	***d***
Basal		1.77	1.04	1.74	0.90	0.27	0.79	0.25
-1 min		2.25	0.93	2.02	1.06	1.22	0.23	0.21
+ 1 min		3.15	1.13	3.77	2.22	0.42	0.68	0.24
+ 5 min		4.68	2.69	5.26	3.44	0.22	0.83	0.29
+ 10 min		6.36	4.52	6.67	4.76	0.03	0.98	0.35
+ 20 min		6.24	4.20	5.91	4.37	0.62	0.54	0.36
+ 40 min		3.99	2.28	3.02	1.74	1.87	0.07	0.27
+ 60 min**		2.78	1.27	1.93	1.05	2.78	< 0.01	0.24
						***U***	***p***	***r***
AUCg		322.29	155.95	307.05	175.82	445	0.62	0.06
AUCi		157.72	144.20	163.45	154.76	475.50	0.95	0.01
		4.52	4.57	4.78	4.71	419.50	0.81	0.03
		4.23	3.75	4.54	3.38	371	0.58	0.07
		2.51	2.74	3.12	3.74	412	0.73	0.05
*		0.56	0.16	0.65	0.11	276	0.04	0.27
**Oxytocin (in pg/ml)**		***M***	***SD***	***M***	***SD***	***t***	***p***	***d***
Basal		1.20	0.21	1.13	0.25	1.20	0.24	0.08
-1 min		1.15	0.19	1.16	0.20	0.10	0.92	0.07
+ 1 min		1.30	0.16	1.33	0.21	0.61	0.55	0.06
+ 5 min		1.30	0.18	1.37	0.26	1.17	0.25	0.07
+ 10 min		1.34	0.30	1.26	0.22	0.98	0.33	0.09
+ 20 min		1.25	0.19	1.25	0.25	0.18	0.86	0.08
+ 40 min		1.21	0.20	1.18	0.23	0.69	0.49	0.08
+ 60 min		1.20	0.24	1.16	0.23	0.63	0.53	0.09
						***U***	***p***	***r***
AUCg		96.21	13.31	97.14	14.48	445	0.77	0.04
AUCi		11.50	9.68	8.52	7.29	393.50	0.30	0.13
		0.34	0.24	0.31	0.13	431	0.78	0.04
		0.35	0.20	0.37	0.17	408.50	0.69	0.05
		0.32	0.24	0.28	0.14	425	0.71	0.05
		0.23	0.11	0.25	0.10	416	0.77	0.04
**Heart rate (in bpm)**		***M***	***SD***	***M***	***SD***	***t***	***p***	***d***
HRT1 -15 min to -1 min		91.50	12.17	87.15	9.78	1.57	0.12	11.02
HRT2 TSST		111.07	12.90	112.34	16.88	0.34	0.74	15.06
HRT3 + 1 min to + 15 min		94.81	15.49	88.66	11.29	1.81	0.08	13.52
HRT4 + 45 min to + 60 min		89.54	16.12	83.80	11.27	1.64	0.11	13.87

German secondary schools were categorized according to their educational track: lower secondary (Mittelschule, 9 years of elementary school), intermediate secondary (Realschule, 6 years of school after 4 years of elementary school), and academic/college-preparatory secondary (Gymnasium, higher level education, 8–9 years of school after 4 years of elementary school); BAI, Beck Anxiety Inventory; BDI-II, Beck Depression Inventory-II; SPAIK, Social Phobia and Anxiety Inventory for Children; ICD-10, International Statistical Classification of Diseases and Related Health Problems 10th Revision; AUCg, Area under the curve with respect to ground; AUCi, Area under the curve with respect to increase; CORT, cortisol; OXT, oxytocin; I, increase; R recovery; abs, absolute change; r, relative change; HRT, Heart rate; bpm, beats per minute; Multiple anxiety diagnoses per person were possible; Sex and gender overlapped in the sample; ^1^Comparisons were conducted using Mann-Whitney-U-Test or t-test, *p* values indicate significant differences in the variables between both groups: * *p* < 0.05; ** *p* < 0.01; *** *p* < 0.001.

### Procedure

The study comprised two 2.5-hour appointments at the Clinic for Child and Adolescent Psychiatry, Psychosomatics, and Psychotherapy at the University of Regensburg, Germany. Participants were screened for eligibility at the first appointment (T1) and characterized by semi-structured interviews and self-reported questionnaires. At the second appointment (T2), participants were exposed to a psychosocial stressor via the TSST, with pre- and post-stress salivary hormone (oxytocin and cortisol) measurements. Physiological (heart rate) and psychometric (perceived anxiety and stress) parameters were also assessed. Participants received a 50€ gift voucher upon study completion.

Participants with anxiety disorder who met the inclusion criteria were assigned to the anxiety group and matched with healthy controls according to educational status. To minimize the effect of age and pubertal development on hormone release^34^, further matching criteria were sex, age, and pubertal status examined using the German version^35^ of the Pubertal Development Scale.

Since children, especially those with anxiety disorders, might already be excited at the laboratory even though the TSST has not started yet, we measured one saliva sample at home in a relaxing atmosphere. Following T1, participants were instructed both verbally and in writing how to collect one saliva sample at home (OXT1/CORT1) and evaluate their mood on a Visual Analogue Scale (VAS1) (for more details see^25^ at 4:00 pm in a relaxed atmosphere, at least 1 h after eating, and not after physical exercise or stress. They were asked to store it in the freezer and bring it to T2 to the laboratory. This established the salivary baseline oxytocin and cortisol levels before the experimental test session (T2) with the TSST. At the laboratory, T2 was also scheduled at 4:00 pm to minimize circadian cortisol rhythm effects^36^. Female participants were tested in the luteal phase of their menstrual cycle^37^. To minimize external effects, participants were instructed not to eat, drink (except water), or smoke 1 h before T2 and during the test session and not to consume drugs and alcohol for at least 1 week before T2. T2 started with a 60-minute relaxation period with relaxing material (e.g., for painting, reading) to minimize previous stress or physical activity effects. After 1 h (1 min before the TSST), the first saliva sample (OXT2/CORT2) was collected and the subjective experience of stress and anxiety was estimated (VAS2). In a second room, after a short task introduction and answering VAS3, psychosocial stress was induced via the TSST. The participants then returned to the previous relaxation room. They were asked to take a seat and calm down by themselves. No relaxation materials were provided to investigate the individual recovery progress. The use of mobile phones was prohibited. During the relaxation period, saliva samples were collected 1, 5, 10, 20, 40, and 60 min post-TSST (OXT3 to OXT8/CORT3 to CORT8) by the experimenter, along with mood assessments via VAS. At the end of the relaxation period, participants received positive feedback and were informed that no video recording was made^33^. The Ecg-data was recorded continuously during T2 using the EcgMove 4 activity sensor (movisens, Germany). From that, the heart rate was calculated using the software DataAnalyzer Base and DataAnalyzer Modul Cardio (Version 1.13.5 (18. June 2019), movisens GmbH, Karlsruhe, Germany, https://www.movisens.com/de/produkte/dataanalyzer-2/), with mean values taken at four 15-minute intervals: before, during, and after the TSST, and at the end of recovery.

### Psychosocial stress task

The TSST^17^ is a well-established tool to induce psychosocial and physiological stress in laboratory settings^38^. It comprises 5 min of free speech and a 5-minute arithmetic task in front of two unfamiliar auditors. Participants are given 5 min to prepare. During the speech task, participants are instructed to convince the audience that they were ideal for the student representative role, believing their performance will be recorded and evaluated. The arithmetic task involves age-adapted mental calculations, subtracting the number 7 (for 11-year-olds) or 13 (for 12- to 18-year-olds) serially from a starting number.

### Psychological measures

At T1, psychiatric diagnoses were assessed by the Mini-International Neuropsychiatric Interview for Children and Adolescents (M.I.N.I.-KID)^39^, a structured interview for axis I disorders according to DSM-IV and ICD-10. The M.I.N.I-KID was performed by two trained study personnel and reviewed by a child and adolescent psychiatry specialist. To assess the severity of psychopathology, dimensional measures were included. Anxiety symptomatology was measured with the Beck Anxiety Inventory (BAI)^40^ and the Social Phobia and Anxiety Inventory for Children (SPAIK)^41^. Depressive symptoms were measured using the Beck Depression Inventory (BDI-II)^42^. As the BDI-II was designed for ages ≥ 13 years, we excluded item 21 (“loss of interest in sex”) from the assessment because it is less applicable to younger participants. For missing data on the 21st BDI variable, we substituted the mean of the available 20 items and included this in the overall BDI total score.

**Fig. 1:**
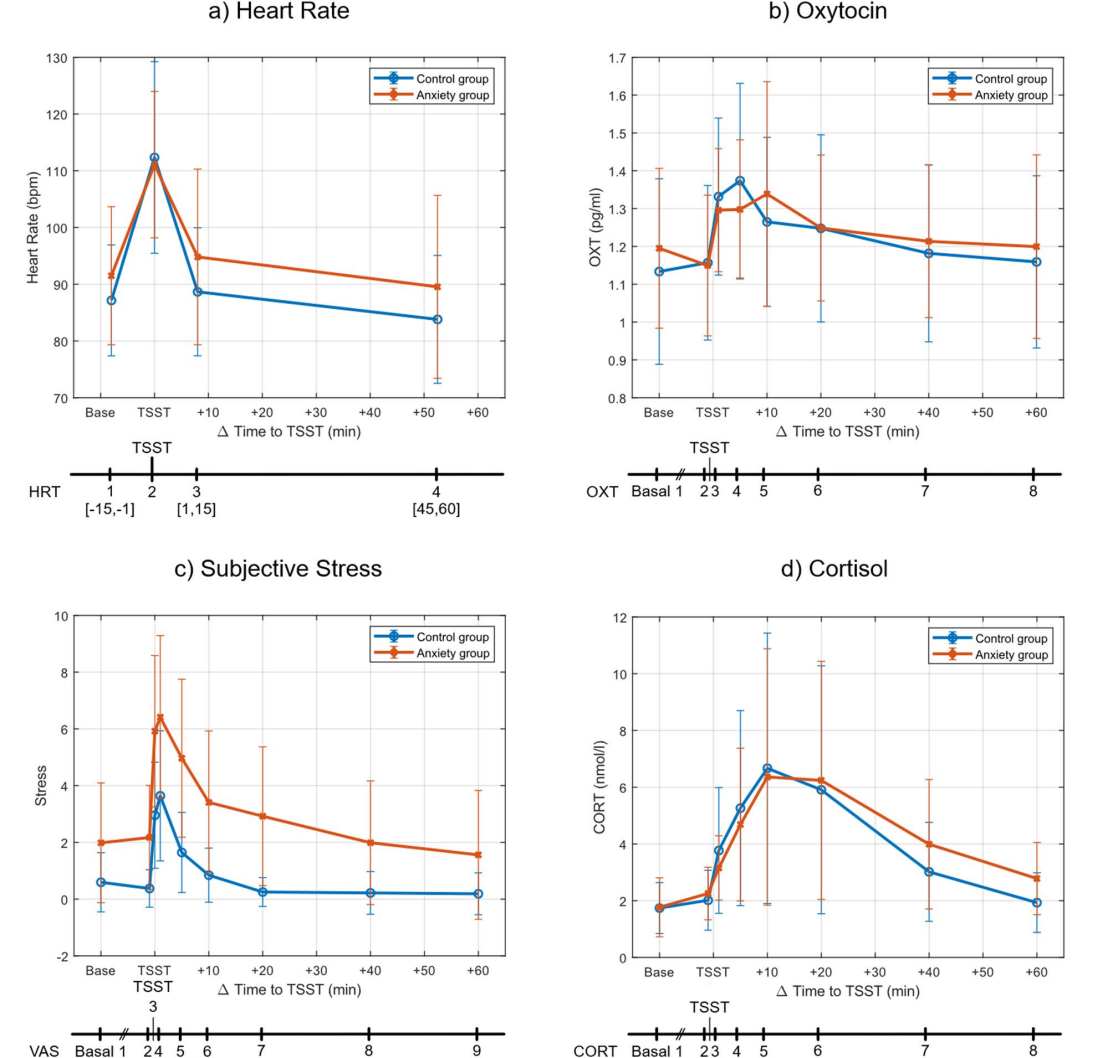
Physiological (heart rate, oxytocin, cortisol) and subjective stress responses (VAS stress) with the timeline of data collection. Parameters monitored under basal conditions and in response to the TSST in children and adolescents with anxiety disorders and healthy controls (age 11–18 years). Control = Control group of healthy participants; Anxiety group = Index group of participants suffering from anxiety disorder; TSST = Trier Social Stress Test; HRT = Heart Rate; [] = Time Interval in minutes to TSST; OXT = Salivary oxytocin level; CORT = Salivary cortisol level; VAS = Visual Analogue Scale stress; Basal = Salivary level at home; pg/ml = picogram per milliliter; nmol/l = nanomole per liter. Data are shown as mean values with standard deviation.

During T2, participants rated their feelings (anxiety and stress) on a VAS at nine time points^25^: at home (VAS1), before the TSST (-1 min, VAS2), during the TSST (0 min, VAS3), and 1, 5, 10, 20, 40, and 60 min post-TSST (VAS4 to VAS9), coinciding with hormonal measures (Fig. 1). VAS scores ranged from 0 (not feeling anxious/stressed/tense/insecure at all) to 100 (feeling highly anxious/stressed/tense/insecure).

### Neuroendocrine measures

Saliva was collected with a cotton swab using a salivette (Sarstedt, Germany). Participants kept the swab in their mouth for 1–2 min and returned it with the saliva to the salivette. Salivettes were stored at − 20 °C until biochemical analysis.

Oxytocin concentrations were quantified by radioimmunoassay at an external laboratory (RIAgnosis, Sinzing, Germany) following established protocols^16^. Each sample underwent evaporation of 300 µl of saliva. Assay sensitivity was within the 0.1 pg/sample range, with intra- and inter-assay variability < 10% and insignificant cross-reactivity with related peptides.

After quantifying salivary oxytocin, the same salivettes were assayed at the Department of Biopsychology, Technical University of Dresden, Germany, for free salivary cortisol levels. Samples were stored at -20 °C until analysis, then thawed and centrifuged at 3000 rpm for 5 min. From the clear supernatant of low viscosity 200 µl were taken and analyzed using a chemiluminescence immunoassay (IBL International, Hamburg, Germany) with high sensitivity. The intra- and inter-assay variability were < 9%.

### Statistical analysis

Statistical analyses and data processing were performed using IBM SPSS version 28.0 (IBM Corp. Armonk, NY, USA). Group comparisons utilized independent *t*-tests for normally distributed data or Mann-Whitney *U* tests otherwise. The anxiety disorder group was analyzed as a transdiagnostic clinical group, as the study was not powered to investigate disorder-specific subtypes. Logarithmic transformation was applied to achieve normal distribution for oxytocin and cortisol analyses. Tables and figures present non-transformed data for ease of interpretation. Outliers were detected by the median absolute deviation (MAD) with a threshold of 3.0^43^. Repeated measures ANOVAs (rmANOVAs) examined stress reactions of oxytocin, cortisol, VAS ratings, and heart rate, with time as the within-subject factor and group and sex as between-subject factors. Sex was included in these models to account for potential confounding effects, as the final sample differed between groups in sex distribution. Additional analyses for females considered contraceptive use. Differences in oxytocin and cortisol reactivity (*p* < 0.05) were followed by post hoc Bonferroni-corrected comparisons. Cortisol responders were detected by a ≥ 15.5% baseline-to-peak increase^44^. To the best of our knowledge, no comparable cut-off for oxytocin increase was defined by previous studies.

Cortisol and oxytocin reactivity were assessed using the area under the curve with respect to ground (AUCg) and increase (AUCi), calculated using the trapezoid formula^45^. Missing values were replaced with group means. Negative AUCi values, resulting from higher hormone concentrations during baseline than after stress exposure, were set to 0^45^. If more than two measures were missing, the AUCi and AUCg were excluded from analyses.

The baseline-to-peak increase was operationalized as a change score (∆OXT_I_abs_, ∆CORT_I_abs_) by subtracting the baseline value (OXT2, CORT2) from the highest post-stress value. A percentage score (increase compared to baseline) was calculated as a measure of reactivity (∆OXT_I_rel_, ∆CORT_I_rel_)^44,46,47^. To assess post-stress recovery, we calculated ∆-change scores (∆OXT_R_abs_, ∆CORT_R_abs_) between the highest post-stress values and the lowest values after the peak (OXT6 to OXT8, CORT5 to CORT8). As absolute levels of recovery can be highest post-stress values influenced by the degree of reactivity, relative recovery change (∆OXT_R_rel_, ∆CORT_R_rel_) was calculated by putting the absolute recovery rate in relation to the highest post-stress values^48^. Group comparisons of basal oxytocin and cortisol levels, AUC, increase and recovery, and relative increase and recovery were performed using Mann-Whitney *U* tests.

FDR-corrected bi-variate Kendall’s τ-correlations were calculated between oxytocin, cortisol, VAS ratings (stress, anxiety), anxiety (BAI), social anxiety (SPAIK), and depression (BDI-II)^49^. Additional correlations were computed between OXT2, CORT2, AUC, absolute increase and recovery, relative increase and recovery, and VAS ratings.

We performed multiple linear regression analyses with ∆CORT_R_rel_ as the dependent variable and ∆CORT_I_rel_, pubertal status, sex, and VAS7 as predictors. Pubertal status and sex were included in this model to examine their potential influence on cortisol recovery, given the group differences in these variables. Further regressions were carried out with CORT3 or CORT6-8 as dependent variables and OXT2 as a predictor.

An a priori power calculation (*n* = 64) was based on data from Bernhard et al.^21^ and was previously published by Goetz et al.^33^. Power analyses were originally conducted for both an rmMANOVA and an rmANOVA. When assuming a correlation between cortisol and oxytocin—as reported by Bernhard et al.^21^—the appropriate model was an rmMANOVA. To achieve a sufficient power of 80%, a sample size of *n* = 30 was estimated. When assuming no correlation, a univariate rmANOVA was considered. As the study also planned a separate MANOVA to assess group × sex effects on oxytocin outcomes, this analysis required a larger sample of *n* = 64, which was adopted as the final target sample size. Accordingly, although the final analyses reported in the manuscript rely on rmANOVAs, the study was powered based on the more conservative MANOVA requirement. Effect sizes are reported using Cohen’s *d*, *r*, or $\:{\eta\:}_{p}^{2}$ with 0.2 being considered a small, 0.5 a medium, and 0.8 a large effect.

## Results

### Sample characteristics

Initially, 67 participants were recruited. Two dropped out after T1 for personal reasons, and one healthy control was excluded after T2 due to reported drug use before the measurements. Thus, 64 participants (34 females (53,1%), 30 males (46,9%); age range 11–18 years; *M*_*age*_=15.06 years; *SD* = 1.75) completed the study and were included in the analysis. Thirty-two participants were assigned to either the anxiety group (21 females (65,6%), 11 males (34.4%)) or the control group (13 females (40,6%), 19 males (59,4%)) according to the inclusion criteria. At the time of data collection, four participants were taking hormonal contraceptives. Ten patients reported using psychotropic medication (*n* = 7 fluoxetine, *n* = 2 melatonin, *n* = 1 isotretinoin). All participants were born in Germany and of white ethnicity. Detailed sample characteristics are described in Table 1.

### Stress response

Psychosocial stress response was successfully induced by the TSST in both groups, validating the stress paradigm. The particular stress responses over time are presented in Fig. 1. The results of the rmANOVAs across the different stress parameters are shown in Table 2.

**Table 2:** Results of the rmANOVA – Validation of the stress paradigm.

	Time			Time x Group			Time x Sex			Time x Group x Sex		
	F (df1,df2)	*p*	η²_*p*_	F (df1,df2)	*p*	η²_*p*_	F (df1,df2)	*p*	η²_*p*_	F (df1,df2)	*p*	η²_*p*_
Cortisol (log)*	43.90 (1.88, 88.22)	< 0.001	0.48	2.38 (1.88, 88.22)	0.10	0.05	3.75 (1.88, 88.22)	0.03	0.07	1.34 (1.88, 88.22)	0.27	0.03
Oxytocin (log)*	11.44 (4.01, 212.37)	< 0.001	0.18	1.32 (4.01, 212.37)	0.26	0.02	0.84 (4.01, 212.37)	0.50	0.02	0.61 (4.01, 212.37)	0.66	0.01
VAS (anxiety)	46.92 (3.12, 165.20)	< 0.001	0.47	9.69 (3.12, 165.20)	< 0.001	0.16	1.01 (3.12, 165.20)	0.39	0.02	1.70 (3.12, 165.20)	0.17	0.03
VAS (stress)	77.21 (3.62, 191.77)	< 0.001	0.59	3.78 (3.62, 191.77)	< 0.01	0.07	1.03 (3.62, 191.77)	0.39	0.02	0.72 (3.62, 191.77)	0.57	0.01
Heart rate*	93.10 (2.55, 150.53)	< 0.001	0.61	3.69 (2.55, 150.53)	0.02	0.06	4.23 (2.55, 150.53)	0.01	0.07	0.27 (2.55, 150.53)	0.82	0.00

Log: Logarithmized values; VAS: Visual Analogue Scale.

*No significant effects for group, sex and group*sex as between-subject factors were found.

VAS anxiety: Significant effects for group as between-subject factor F(1,53) = 30.79, *p* < 0.001, η²_p_ = 0.37.

VAS stress: Significant effects for group as between-subject factor F(1,53) = 33.45, *p* < 0.001, η²_p_ = 0.39.

Accordingly, there was a significant main effect of time for all stress parameters (cortisol, oxytocin, heart rate, subjective levels of anxiety/stress). A significant main effect of group was found for the subjective levels of anxiety/stress, with the anxiety group showing significantly higher levels of perceived anxiety/stress at all time points. Time×group interactions were found for all subjective levels, indicating that the anxiety group showed a pronounced early increase in anxiety/stress levels followed by a gradual decline over time, whereas the control group exhibited consistently lower scores and more rapid decreases to near zero levels at later time points. Furthermore, a time×group interaction for heart rate was found with a rapid in- and decrease of heart rate by the control group. However, the heart rate itself did not significantly differ between the groups (see Table 1). Time×sex interactions were observed for cortisol and heart rate with higher heart rate in females during the TSST (*t*(61) = 2.34, *p* = 0.02, *d* = 14.44) and absolute lower cortisol levels before stress exposure in females, and higher cortisol levels after stress exposure (for details, see Table 2).

#### Oxytocin

As expected, a significant salivary oxytocin response to the TSST occurred with a significant main effect of time (*F*(4.01, 212.37) = 11.44, *p* < 0.001, $\:{\eta}_{\mathrm{p}}^{\mathrm{2}}$ =0.18). However, no interaction effect of time×group was found (*F*(4.01, 212.37) = 1.32, *p* = 0.26, $\:{\eta}_{\mathrm{p}}^{\mathrm{2}}$ =0.02). Different than expected, no group differences were found in OXT1 (*t*(59) = 1.20, *p* = 0.24, *d* = 0.08) and OXT2 before stress (*t*(60) = 0.10, *p* = 0.92, *d* = 0.07).

#### Cortisol

Overall, 84.4% of the participants exhibited a substantial stress response in terms of cortisol, with a baseline-to-peak cortisol release ≥ 15.5%. The responses of two participants could not be calculated because of missing values. All participants (responders and non-responders) were included in the statistical analyses.

Concerning cortisol release, we found no significant group differences in CORT1 (*t*(56) = 0.27, *p* = 0.79, *d* = 0.25) and CORT2 (*t*(60) = 1.22, *p* = 0.23, *d* = 0.21). However, the anxiety group had significantly higher levels of CORT8 than the control group (*t*(57) = 2.78, *p* < 0.01, *d* = 0.24). No group differences in cortisol were observed at other time points. Healthy controls had a significantly higher ∆CORT_R_rel_ (*U* = 276, *p* = 0.04, *r* = 0.27) than the anxiety group.

Mean values of oxytocin and cortisol indices are presented in Table 1.

#### Subjective parameters (stress/anxiety)

The subjective level of stress (sum of VAS ratings) was rated higher by the anxiety group (*M* = 26.57, *SD* = 12.82) than by the controls (*M* = 9.93, *SD* = 5.88; *U* = 100.50, *p* < 0.001, *r* = 0.64). Anxiety levels were also rated higher by the anxiety group (*M* = 19.32, *SD* = 12.85) than the controls (*M* = 4.27, S*D* = 4.65; *U* = 105, *p* < 0.001, *r* = 0.64). The anxiety group reported significantly higher subjective stress and anxiety levels than the controls across all time points (see Fig. 1).

#### Supplementary analyses

Taking the effect of contraceptives on hormonal release into account, in a subsidiary analysis, it was found that females who were not taking hormonal contraceptives had significantly higher levels of OXT2 (*M* = 1.18, *SD* = 0.18) than females taking hormonal contraceptives (*M* = 1.04, *SD* = 0.01; *t*(31) = 4.01, *p* < 0.001, *d* = 0.06). For CORT1 and CORT2, no group differences were found between females who were taking and females who were not taking contraceptives. We did not observe interaction effects of time×contraceptives in females for cortisol (*F*(1.90,45.58) = 3.07, *p* = 0.06, $\:{\eta}_{\mathrm{p}}^{2}$ =0.11) or the oxytocin response (*F*(3.71,100.12) = 0.39, *p* = 0.80, $\:{\eta}_{\mathrm{p}}^{2}$ =0.01).

### Correlation of cortisol, oxytocin, and subjective stress

Across all participants, OXT2 correlated positively with cortisol levels at 1 min (τ(54) = 0.24, *p* = 0.01), 20 min (τ(59) = 0.22, *p* = 0.02), 40 min (τ(57) = 0.27, *p* = 0.03), and 60 min (τ(55) = 0.21, *p* = 0.02) post-TSST (Table 3). To examine potential confounding effects, pubertal status was correlated with endocrine and physiological measures. Pubertal status positively correlated with CORT7 (τ(59) = 0.21, *p* = 0.03), CORT8 (τ(57) = 0.27, *p* = 0.01), OXT4 (τ(58) = 0.20, *p* = 0.05), and HRT2 (τ(61) = 0.23, *p* = 0.02), but negatively with ∆CORT_R_rel_ (τ(55)=-2.77, *p* < 0.01).

**Table 3:** Bivariate correlations for oxytocin and cortisol levels.

	CORT1	CORT2	CORT3	CORT4	CORT5	CORT6	CORT7	CORT8	OXT1	OXT2	OXT3	OXT4	OXT5	OXT6	OXT7	OXT8
CORT1 basal	-															
CORT2 -1 min	0.024	-														
CORT3 + 1 min	0.018	0.328***	-													
CORT4 + 5 min	0.035	0.122	0.723***	-												
CORT5 + 10 min	0.026	0.066	0.653***	0.848***	-											
CORT6 + 20 min	0.003	0.097	0.585***	0.716***	0.823***	-										
CORT7 + 40 min	0.005	0.127	0.584***	0.645***	0.711***	0.791***	-									
CORT8 + 60 min	− 0.017	0.158	0.475***	0.525***	0.567***	0.662***	0.805***	-								
OXT1 basal	0.069	0.028	0.026	− 0.061	− 0.041	− 0.009	0.023	0.039	-							
OXT2 -1 min	0.050	0.117	0.239*	0.115	0.148	0.216*	0.199*	0.214*	0.470***	-						
OXT3 + 1 min	0.012	0.077	0.134	0.054	0.083	0.092	0.041	0.004	0.434***	0.554***	-					
OXT4 + 5 min	0.050	− 0.018	0.114	0.100	0.103	0.109	0.038	0.018	0.203*	0.299***	0.331***	-				
OXT5 + 10 min	− 0.061	− 0.069	0.084	0.054	0.047	0.088	0.057	0.088	0.156	0.162	0.246**	0.575***	-			
OXT6 + 20 min	− 0.120	− 0.062	0.044	0.050	− 0.031	0.025	− 0.051	− 0.039	0.176*	0.163	0.207*	0.418***	0.463***	-		
OXT7 + 40 min	− 0.023	− 0.108	0.174	0.194*	0.130	0.157	0.096	0.123	0.183*	0.261**	0.244**	0.299***	0.363***	0.547***	-	
OXT8 + 60 min	0.014	− 0.015	0.235*	0.197*	0.108	0.132	0.072	0.112	0.146	0.276**	0.257**	0.309***	0.400***	0.480***	0.583***	-

Correlation coefficients correspond to Kendall’s τ. CORT = cortisol, OXT = oxytocin, min = minute. * *p* < 0.05; ** *p* < 0.01; *** *p* < 0.001 (2-tailed).

In a regression model examining the association between OXT2 before stress exposure and the cortisol levels after stress exposure, OXT2 predicted CORT3 (*F*(1,54) = 6.10, *p* = 0.02, R^2^ = 0.10, β = 0.32, *t* = 2.47), CORT6 (*F*(1,59) = 5.89, *p* = 0.02, R^2^ = 0.09, β = 0.30, *t* = 2.43), CORT7 (*F*(1,57) = 4.25, *p* = 0.04, R^2^ = 0.07, β = 0.26, *t* = 2.06), and CORT8 (*F*(1,55) = 4.50, *p* = 0.04, R^2^ = 0.08, β = 0.28, *t* = 2.12).

It was hypothesized that lower oxytocin release after stress correlates with a higher increase in cortisol and higher subjective stress response. We observed, that ∆OXT_I_abs_ did not significantly correlate with ∆CORT_I_abs_ (τ(53) = 0.01, *p* = 0.90), but correlated positively with ∆CORT_R_rel_ (τ(51) = 0.20, *p* = 0.04).

Higher levels of ∆OXT_R_abs_ were associated with lower subjective stress levels during recovery (20 min: τ(56)=-0.24, *p* = 0.01; 40 min: τ(57)=-0.26, *p* = 0.01; 60 min: τ(57)=-0.26, *p* = 0.01). ∆CORT_R_rel_ also negatively correlated with the subjective stress level at the end of the recovery period (10 min: τ(54)=-0.20, *p* = 0.04; 20 min: τ(55)=-0.40, *p* < 0.001; 40 min: τ(55)=-0.39, *p* < 0.001; 60 min: τ(55)=-0.31, *p* < 0.01; Table 4).

**Table 4:** Bivariate correlations for increase, recovery and subjective levels of stress.

	1	2	3	4	5	6	7	8	VAS6	VAS7	VAS8	VAS9
1 Delta increase OXT	-											
2 Delta recovery OXT	0.227^*^	-										
3 Delta increase percentage OXT	0.842^***^	0.144	-									
4 Delta recovery percentage OXT	0.126	0.812^***^	0.105	-								
5 Delta increase CORT	0.012	0.012	− 0.028	− 0.034	-							
6 Delta recovery CORT	0.062	0.050	0.033	0.020	0.780^***^	-						
7 Delta increase percentage CORT	0.030	− 0.060	− 0.011	− 0.094	0.787^***^	0.606^***^	-					
8 Delta recovery percentage CORT	0.196^*^	0.034	0.174	− 0.001	0.413^***^	0.543^***^	0.379^***^	-				
VAS6 stress + 10 min	− 0.128	− 0.136	− 0.110	− 0.108	− 0.104	− 0.099	− 0.122	− 0.199^*^	-			
VAS7 stress + 20 min	− 0.152	− 0.244^*^	− 0.104	− 0.168	− 0.226^*^	− 0.277^**^	− 0.207^*^	− 0.398^***^	0.726^***^	-		
VAS8 stress + 40 min	− 0.155	− 0.260^*^	− 0.140	− 0.225^*^	− 0.183	− 0.266^**^	− 0.146	− 0.391^***^	0.589^***^	0.729^***^	-	
VAS9 stress + 60 min	− 0.123	− 0.255^*^	− 0.110	− 0.246^*^	− 0.141	− 0.187	− 0.134	− 0.311^**^	0.498^***^	0.655^***^	0.805^***^	-

Correlation coefficients correspond to Kendall’s τ. CORT = cortisol, OXT = oxytocin, min = minute, VAS = visual analogue scale stress.

* *p* < 0.05; ** *p* < 0.01; *** *p* < 0.001 (2-tailed).

CORT8 showed small positive correlations with social anxiety on the SPAIK (τ(57) = 0.24, *p* = 0.01), anxiety on the BAI (τ(57) = 0.21, *p* = 0.02), and depression on the BDI-II (τ(57) = 0.24, *p* = 0.01). Depression on the BDI-II negatively correlated with ∆CORT_R_rel_ (τ(55)=-2.14, *p* = 0.02).

A multiple regression predicted ∆CORT_R_rel_ from ∆CORT_I_rel_, pubertal status, sex, and the subjective level of stress on the VAS7. ∆CORT_I_rel_ and VAS7 significantly predicted ∆CORT_R_rel_ (*F*(4,51) = 13.71, *p* < 0.001, R^2^ = 0.52). ∆CORT_I_rel_ (β = 0.47, *t* = 4.56, *p* < 0.001) and VAS7 (β=-0.36, *t*=-3.23, *p* < 0.001) significantly added to the prediction, but pubertal status and sex did not.

### Potential confounding variables

Because the groups differed in sex and pubertal status (Table 1), these variables were included in the primary statistical models. Sex was incorporated as a between-subject factor in all rmANOVAs examining endocrine and physiological stress responses. Although time × sex interactions were observed for cortisol and heart rate, no sex × group interaction emerged for cortisol or oxytocin responses. Pubertal status showed small correlations with some endocrine measures during recovery but did not significantly predict relative cortisol recovery in the regression analysis. Overall, inclusion of sex and pubertal status did not alter the pattern of group differences.

## Discussion

This study examined the endocrinological (salivary oxytocin and cortisol), subjective (anxiety and stress), and physiological (heart rate) responses to acute psychosocial stress in children and adolescents with anxiety disorders compared to healthy controls. We hypothesized that the clinical sample would show a lower basal oxytocin level before stress exposure and less oxytocin reactivity than healthy controls after psychosocial stress induction. Unexpectedly, basal and reactive oxytocin levels after psychosocial stress induction did not significantly differ between the groups. Consistent with previous studies, oxytocin levels in saliva increased after psychosocial stress exposure in the non-clinical sample^16,18–21^. In addition, we found a comparable and significant increase in oxytocin levels after TSST exposure in children and adolescents suffering from anxiety disorders. Different from the hypothesis, basal levels of oxytocin and the stress-induced increase in oxytocin did not significantly differ between healthy participants and patients with anxiety disorders. The result that no group differences were found supports oxytocin as a biomarker of the stress response^8,10–12^ in children and adolescents regardless of the presence of anxiety disorders. In our study, adolescents with anxiety disorders and healthy controls did not differ in terms of cortisol reaction over time, which is in line with previous findings^32,50^. While adolescents with borderline personality disorder did not differ from healthy controls in terms of cortisol stress response^24^, youth with conduct disorder or oppositional defiant disorder showed blunted cortisol response^51^. In a review by Bernhard et al., in youths with depression disorder, higher as well as lower cortisol responses were observed, which may be influenced by sex differences^51^. In children with attention-deficit hyperactivity disorder, the cortisol response was driven by comorbidities and subtypes. Taken together, the reasons that no group difference between the anxiety and control group in the endocrinological stress reaction over time were found remain speculative, but may be influenced by comorbidities, age and sex. The TSST generates a subjective stress response with a significant increase in subjective stress ratings^20,21,50^. As expected, the anxiety group reported higher subjective stress and anxiety at all time points. The discrepant physiological and psychological stress response was also found in other studies with adolescents suffering from anxiety^52,53^ and depressive disorders^22^. Since adolescents with externalizing behaviour did not differ from healthy controls in their psychological stress response than healthy controls^53^, Bernhard et al. hypothesized that the discrepancy between psychological and neuroendocrine stress may be a specific characteristic of internalizing rather than externalizing disorders^22^.

We further hypothesized that oxytocin release after stress would negatively correlate with the increase in cortisol and the subjective stress response. In our study, oxytocin increase correlated with relative cortisol recovery but not with cortisol increase. Furthermore, pre-stress oxytocin levels positively predicted cortisol levels after stress exposure. In line with a regulatory effect of oxytocin on cortisol^19,26,28,29^, pre-stress oxytocin levels positively predicted cortisol levels during recovery across all participants. However, oxytocin increase did not correlate with cortisol increase or subjective stress. Nonetheless, oxytocin increase positively correlated with relative cortisol recovery as observed in previous studies^18,19,21^, indicating that the stress-regulatory effects of oxytocin are associated with cortisol recovery and not with cortisol increase. These findings support the hypothesis of a recovery-boosting effect of oxytocin rather than a reactivity-buffering function^19^. Reinforcing the importance of recovery, in all patients higher oxytocin recovery and higher relative cortisol recovery were associated with lower subjective stress levels during recovery^18,19^. Furthermore, healthy controls had significantly higher levels of relative cortisol recovery than the anxiety group. In summary, it could be assumed that high baseline oxytocin might result in marginal increases in oxytocin, correlating with less cortisol recovery potential during stress. This, in turn, potentially leads to higher absolute cortisol levels after stress exposure and increased subjective stress. Taken together, the findings suggest that in the pathogenesis of anxiety disorders, it is not merely the oxytocin increase per se that is relevant, but rather the co-regulation between oxytocin, cortisol recovery and the subjective experience of stress.

The third hypothesis was that the levels of perceived feelings of anxiety and stress would differ significantly between both groups and correlate negatively with oxytocin levels. As predicted, participants with anxiety disorder reported significantly more anxiety and stress than controls. However, the increase in oxytocin and cortisol did not correlate with subjective stress levels post-stress. Instead, we found that subjective stress was negatively correlated with oxytocin recovery and relative cortisol recovery. Subjective stress levels during recovery (+ 20 min) significantly predicted the relative cortisol recovery. These findings support the evidence of a covariance between the psychological and physiological stress response during the TSST regarding stress perception in healthy controls^25^ and adults with social anxiety disorder^50^. Interestingly, participants with anxiety disorders exhibited significantly higher cortisol levels at the end of the recovery period (+ 60 min). Across all participants, high levels of cortisol at the end of recovery positively correlated with social anxiety, anxiety, and depression. Further research is needed to explore the regulatory effects of oxytocin on stress recovery and the effects of oxytocin and cortisol recovery on psychiatric disturbances.

Increasing evidence also indicates substantial sex differences in cortisol stress reactivity across psychiatric disorders (e.g.^32^). In the present study, an interaction of time and sex resulted in higher cortisol levels in females after stress exposure. We did not find any time and sex interaction for the oxytocin stress reaction, though previous studies indicated an interaction of oxytocin and sex hormones in anxiety disorders^54^ and globally higher oxytocin levels in females compared to males^55^. We found significantly lower basal oxytocin levels in females using hormonal contraceptives, which is in line with findings from de Jong et al.^16^. In contrast, some others have identified that females who take contraceptives have higher overall oxytocin levels independent of the stress phase^19^ and higher plasma OXT levels^54^ compared to females who do not. However, the sample of contraceptive users in our study was very small (*n* = 4). No significant time and contraceptive interactions were observed on the oxytocin and cortisol stress response. Additional studies with larger samples are needed to investigate and confirm potential interactions with sex hormones and sex differences in the oxytocin stress reaction across psychiatric disorders.

This study has some limitations. First, oxytocin and cortisol concentrations were collected as reliable saliva samples^16^, which primarily reflect peripheral oxytocin release and may differ from central release, potentially affecting sensitivity^11^. Second, given the high comorbidity rate between depression and anxiety disorders, participants with comorbid mild or moderate depressive episodes were included^3^. Although this enhances the study’s ecological validity, depressive symptoms can influence stress reactions, with effects varying by sex^32^, and influence reactivity and recovery slopes^56^. Bernhard et al. showed that adolescents suffering from major depressive disorder showed a lower cortisol and oxytocin stress response than healthy controls^22^. In the present study, depression on the BDI-II correlated negatively with the relative cortisol recovery, highlighting the potential influence of depressive symptoms. Larger studies are needed to better understand the effects of depressive symptoms on oxytocin stress reactions and the interplay between cortisol and oxytocin across psychiatric disorders. Another limitation is that, after recruitment, the matching criteria were not met consistently, resulting in significant differences in pubertal status and sex. However, this study is the first to link pubertal status to oxytocin (at 5 min) and cortisol (at 40 min and 60 min) release after stress and relative cortisol recovery. Pubertal status did not predict cortisol recovery, and whether oxytocin reactions differ by pubertal stage is unclear. Future studies should independently replicate these findings and examine variations in oxytocin release across pubertal development. Further limitation is that some participants in the anxiety disorder group were taking psychotropic medication. Due to the small and heterogeneous medicated subgroup, medication effects were not analyzed separately. Consequently, potential influences of medication on neuroendocrine stress responses cannot be ruled out and should be addressed in future studies with larger samples. As children, particularly those with anxiety disorders, may already be excited at the laboratory before the TSST begins, we collected one saliva sample at home in a relaxed setting. On one hand, it is a study’s strength that we measured a baseline oxytocin and cortisol as well as subjective stress level at home (OXT1/CORT1/VAS1) in a relaxing atmosphere not influenced by the clinical environment. However, on the other hand, the comparability to all other times points is limited, because the collection was unmonitored and external stressors were not assed. Therefore, for OXT1/CORT1/VAS1, only group comparisons and correlations were applied. For calculating ∆oxytoxin/cortisol increase/recovery, AUC, rmANOVA we used OXT2/CORT2, measured at the laboratory, as a baseline.

The strengths of this study include detailed participant characterization and the inclusion of possible hormone release covariates^57^, such as pubertal status, medication, age, menstrual cycle, and sex in the analyses. Both groups were well-matched in education, intelligence, and age. Compared to previous studies^57^, this study assessed salivary hormone levels with a finer time resolution at eight time points at home, before and after stress exposure, and even addressed recovery.

## Conclusion

This study is the first to investigate the oxytocin stress response in a clinical sample of children and adolescents with anxiety disorders. The participants with anxiety disorders and healthy controls did not differ in basal oxytocin levels and responses after stress exposure. Overall, oxytocin emerged as a reliable stress marker unaffected by the presence of anxiety disorders. Thus, oxytocin should be investigated as a general stress hormone alongside cortisol in social stress tests. We provide evidence that oxytocin plays an important role in cortisol and psychological stress recovery rather than reactivity. These results justify further investigations into the interplay between oxytocin and cortisol recovery across different psychiatric disorders, ages, and pubertal stages.

## Data Availability

The datasets generated during and/or analysed during the current study supporting the conclusions of this article are available by the corresponding author on reasonable request.
